# SHAPE-MaP-Based Assessment of the Structure of Citrus Tristeza Virus Long Non-Coding RNA

**DOI:** 10.3390/v18040470

**Published:** 2026-04-16

**Authors:** Arianna Spellman-Kruse, Jodi L. Bubenik, Tathiana Ferreira Sa Antunes, Alexander J. Lawrence, Maurice S. Swanson, Ying Wang, Svetlana Y. Folimonova

**Affiliations:** 1 Department of Plant Pathology, Institute of Food and Agricultural Sciences, University of Florida, Gainesville, FL 32611, USA; arianna.kruse@usda.gov (A.S.-K.); tathianafsantunes@gmail.com (T.F.S.A.); alawrence1@ufl.edu (A.J.L.); ying.wang1@ufl.edu (Y.W.); 2 Department of Molecular Genetics & Microbiology, Center for NeuroGenetics and the Genetics Institute, University of Florida, Gainesville, FL 32611, USA; jbubenik@ufl.edu (J.L.B.); mswanson@ufl.edu (M.S.S.)

**Keywords:** citrus tristeza virus, long non-coding RNA, selective 2′-hydroxyl acylation analyzed by primer extension and mutational profiling, RNA structure, virus strains

## Abstract

The 5′-proximal region of the citrus tristeza virus (CTV) RNA genome is a hub where several elements involved in different facets of the virus cycle reside, including the sequences driving the production of the viral long non-coding RNA (lncRNA) LMT1. The sequence of this region is one of the most divergent genome areas, allowing for strain differentiation. Beyond its use in assessing viral population diversity, the region provides a valuable model for studying the conservation of RNA structure and function despite sequence variation. Here, we integrated comparative in silico analysis of the LMT1 region from variants of eight CTV strains with selective 2′-hydroxyl acylation, analyzed by primer extension and mutational profiling (SHAPE-MaP) probing of in vitro–generated LMT1 RNAs from two divergent strains, T36 and T68. The predicted consensus structures revealed 19 putative, conserved stem-loops. The SHAPE-MaP reactivity data supported and substantiated the thermodynamics-based predictions for the 15 previously uncharacterized stem-loops and two functional elements identified earlier. The strong structural conservation across strains highlights that the LMT1 RNA structure contributes to its function during CTV infection. These results provide the first experimentally supported structure of this viral lncRNA and lay the foundation for defining how individual RNA motifs influence CTV biology.

## 1. Introduction

Citrus tristeza virus (CTV; genus *Closterovirus*, family *Closteroviridae*) is the most economically important virus affecting the global citrus industry [1,2]. CTV has multiple genetically distinct strains, including T36, T68, T3, T30, VT, HA16-5, S1, and RB, which comprise variants with considerable phenotypic variability, ranging from asymptomatic to highly virulent [2,3,4,5,6]. In most severe cases, CTV infection can lead to the rapid decline and death of citrus trees, with millions of plants having already been lost worldwide due to disease or preemptive removal [7,8]. Conversely, some mild CTV variants cause no visible symptoms and, due to the amenability of the virus to genetic manipulation, offer potential as vectors for therapeutic delivery and biocontrol of other citrus pathogens [3,7,8,9]. A deeper understanding of the determinants of the pathogenicity and evolution of CTV strains is critical for developing resistant citrus cultivars, optimizing management strategies, and leveraging CTV as a biological tool.

**Figure 1:**
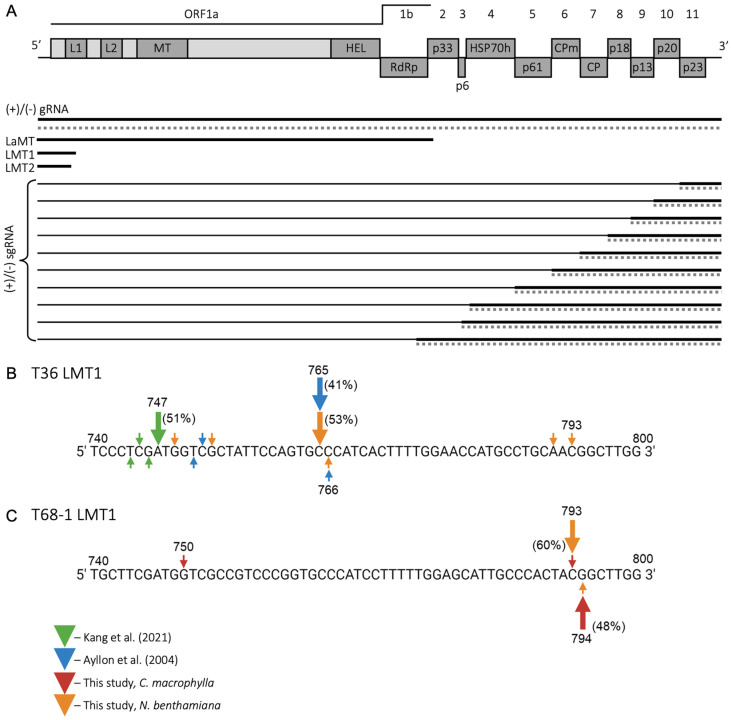
Organization of the CTV genome, subgenomic RNAs produced, and assessment of LMT1 length: (**A**) Scheme of the CTV genome and the genomic (gRNA) and subgenomic RNA (sgRNA) species produced. Boxes indicate the open reading frames or domains encoded in the virus genome. MT, methyltransferase; HEL, helicase; RdRp, RNA-dependent RNA polymerase domains; CPm and CP, minor and major coat proteins. (**B**) Mapping of the T36 LMT1 3′ terminus. Arrows in different colors point to the nucleotide positions corresponding to the 3′ termini of T36 LMT1 as per the respective studies: green for Kang et al. (2021) [19], blue for Ayllon et al. (2004) [20], and orange for sites identified in this study via 3′ Rapid Amplification of cDNA Ends (3′ RACE). For each study, the largest arrow marks the nucleotide position identified as the 3′ terminus of LMT1 in most clones (the number of clones is shown as a percentage). (**C**) Determination of the T68 LMT1 3′ terminus via 3′ RACE. Arrows in red and orange represent the results obtained using virus-infected *Citrus macrophylla* or *Nicotiana benthamiana*, respectively. The largest arrows denote the nucleotide positions identified as the LMT1 terminus in most clones, with the proportion of clones reflecting the respective terminus reported as a percentage.

CTV has a large (19.3 kb) positive (+)-sense RNA genome, encompassing 12 open reading frames (ORFs) that encode proteins involved in different steps of the virus cycle (Figure 1A) [10,11,12,13,14,15,16]. During infection, CTV produces a plethora of distinct RNA species, including the full-length (+)- and (−)-sense genomic RNAs, 3′-coterminal (+)- and (−)-sense subgenomic RNAs (sgRNAs), defective viral genomes, and three 5′-coterminal sgRNAs of (+) polarity—a longer RNA (LaMT) and two shorter low-molecular-weight tristeza RNAs, LMT1 and LMT2 [17] (Figure 1A). The genomic RNA functions as a template for the translation of ORFs 1a and 1b, producing polyproteins needed for virus replication, while the 3′-coterminal (+)-sense sgRNAs drive the expression of the genes positioned in the 3′ half of the CTV genome, encoding proteins participating in the assembly of virus particles, virus movement, and virus interaction with the host [13,14,15,18]. Notably, the 5′-coterminal sgRNAs, LMT1 and LMT2, lack canonical ORFs and are therefore regarded as viral long non-coding RNAs (lncRNAs).

LncRNAs are RNA transcripts of more than 200 nucleotides (nts) in length lacking protein-coding potential, and are abundantly produced by many organisms, including viruses [21]. Numerous studies conducted in recent years have demonstrated that lncRNAs regulate important biological processes, including those involved in development, signaling, disease, and responses to environmental stresses [22]. In the context of viruses, lncRNAs have emerged as key regulators of infection and virus–host interactions. Many viruses that infect animals and plants encode lncRNAs, which influence viral replication, suppress host immune responses, and modulate cellular metabolism to create a more favorable environment for infection [23,24,25]. Several plant viruses encode lncRNAs that counteract the host RNA silencing machinery, a primary antiviral defense mechanism in plants, and thereby enhance viral persistence [26,27].

The CTV lncRNAs LMT1 and LMT2 are of particular interest as they overlap a region in ORF1a, which was denoted as an important identifier region for CTV strain classification [5]. The overall genomic nucleotide identity among CTV strains ranges between 80.3 and 92.4%, with the greatest divergence occurring in the 5′-proximal region, where nucleotide identity ranges between 72.1 and 91.2% [5]. Depending on the CTV strain, the length of LMT1 has been mapped between 747 and 854 nts [19,28], while LMT2 ranges from 630 to 746 nts [28,29]. LMT1 is produced in high amounts, early in infection, while LMT2 accumulates at a later infection stage. The mechanism of the latter RNA production as well as its role in virus infection remains unclear, yet it was shown that the accumulation of LMT2 is associated with virion assembly [17,20,29]. In contrast, LMT1 is a product of termination of the genomic (+)-strand synthesis at a specific controller element on the (−) strand. Functionally, LMT1 RNA has been implicated in counteracting the salicylic acid–mediated plant responses upon viral infection of the host [30]. Importantly, LMT1 was also shown to be crucial for infection of the native citrus host, as CTV mutants unable to produce LMT1 failed to establish infection [30]. Furthermore, the viral multifunctional p33 protein, which is involved in CTV–host interactions as well as the interactions between virus variants, was shown to bind LMT1 at two regions: a region within nts 201 and 400 and between nt 601 and the 3′ terminus of LMT1 [19]. Given the sequence divergence among CTV strains, particularly in the 5′-proximal genomic region, conservation of the RNA secondary/tertiary structure rather than the primary sequence may impact LMT1 function. The defined biogenesis pathway, clear biological relevance, and its essential role in viral infection make LMT1 a meaningful target for secondary structure analysis.

It is well-established that lncRNAs act in association with other viral or host RNAs and key regulatory proteins. As with many RNA molecules, lncRNAs fold into complex structures stabilized by intramolecular base-pairing, forming sub-structures such as hairpins, bulges, pseudoknots, and G-quadruplexes that can serve as scaffolds for macromolecule interactions. Considering that, defining the structure of lncRNAs is a necessary step in determining their mechanisms of action. Research efforts have been made in the profiling of RNA structures, both computationally through machine learning predictions and experimentally through chemical structure probing. Selective 2′-hydroxyl acylation analyzed by primer extension and mutational profiling (SHAPE-MaP) enables high-resolution analysis of RNA flexibility across transcripts and allows incorporation of experimental data into folding predictions [31,32,33]. To date, no CTV-derived RNAs have been chemically probed for secondary structure, and the only CTV RNA structural assessment studies have been performed on individual stem-loops through computer modeling and compensatory mutations.

In this study, using an in silico approach, we examined the primary and secondary structures of LMT1 RNAs produced by CTV variants representing eight different strains. Through comparison of stable and evolutionarily conserved RNA structures, we identified RNA structural motifs in the LMT1 region. Next, to assess the accuracy of the structure predictions and support the in silico analysis, we applied the SHAPE-MaP procedure using in vitro–generated LMT1 RNAs of two CTV variants belonging to two distinct CTV strains, LMT1 RNAs of the T36 and T68-1 variants. We first validated the experimental conditions using T36 LMT1 and confirmed successful RNA generation, modification, and reverse transcription. SHAPE-MaP yielded reproducible, high-resolution data that enabled secondary structure prediction. LMT1 was predicted to fold into a highly structured conformation by multiple thermodynamics-based algorithms, and the SHAPE data were consistent with these predictions. The motifs identified through evolutionary conservation were then compared with the SHAPE-derived data. Overall, many of the putative evolutionarily conserved motifs were reinforced by the in vitro SHAPE-MaP probing, enabling the fine-tuning and improvement of LMT1 structural predictions, as well as identification of RNA elements that may underlie crucial roles of this viral lncRNA in CTV infection and virus–host interactions.

## 2. Materials and Methods

### 2.1. 3′ Rapid Amplification of cDNA Ends of T36 and T68-1 LMT1

To determine the 3′ end of LMT1 RNA produced upon infection with the T36 variant of CTV (GenBank accession # AY170468), *Nicotiana benthamiana* plants infected with an infectious cDNA clone of T36, CTV-T36 [34], were used as a source of the LMT1 T36 RNA template. Inoculation of *N. benthamiana* plants was described previously [35]. Total RNA was extracted using the Direct-zol RNA Miniprep kit (Zymo Research, Irvine, CA, USA), and the RNA integrity was confirmed by agarose/ethidium bromide gel electrophoresis. A poly (A) tail was added to the RNA 3′ end with *Escherichia coli* poly (A) polymerase (Applied Biosystems, Waltham, MA, USA), according to the manufacturer’s instructions. The cDNA synthesis was carried out using SuperScript™ III RT and the GeneRacer Oligo dT primer, following the instructions provided with the GeneRacer kit (Invitrogen, Carlsbad, CA, USA). The 3′ terminus of LMT1 was amplified using the GeneRacer 3′ primer and a gene-specific primer (GSP1, 5′ GTA GTC CTG CAC TAC TTT G 3′) designed from the sequence of the CTV-T36 variant. The purified PCR products were cloned into a pCR^®^-Blunt II-TOPO^®^ vector (Invitrogen, Carlsbad, CA, USA). The plasmid DNA from the 19 generated clones was sequenced, and those sequences were mapped to the known LMT1 sequence for T36 CTV to determine the 3′ ends.

To identify the 3′ end of LMT1 produced by the variant T68-1 of the T68 CTV strain (GenBank accession # JQ965169), *N. benthamiana* plants infected with a hybrid virus CTV-L1L2T68 [36]—engineered based on the T36 cDNA clone in which the region between nts 108 and 3012 was substituted with a cognate region from the T68-1 genome—and *C. macrophylla* plants infected with the wild-type T68-1 were used as the source materials for 3′ RACE. The cDNA synthesis and PCR were conducted as described above using the GeneRacer Oligo dT primer, GeneRacer 3′ primer, and a gene-specific primer (GSP2, 5′ CAG GTC CTC GGC TTT CGC TGT ACG 3′) designed according to the sequence of CTV T68-1. The plasmid DNA from 20 clones harboring the sequence of T68-1 LMT1 was obtained. Those clones were then sequenced, and the length of T68-1 LMT1 was assessed. To further validate the findings from *N. benthamiana*, total RNA extracted from the citrus plants infected with wild-type T68-1 CTV was used as a template for the 3′ RACE procedure, and the 27 generated clones were sequenced and used for 3′ end determination.

### 2.2. Generation of LMT1 Plasmids

Two variations of the T36 LMT1 sequence, one encompassing the 5′-terminal region of the CTV genome between nts 1 and 750 and the other harboring nts 1 to 794, were PCR-amplified using pCTV-T36 as a template [34] and cloned into the pUC19 vector plasmid under the T7 promoter using the restriction endonucleases StuI and EcoRI (New England Biolabs, Ipswich, MA, USA). Sequences of the oligonucleotides used for cloning are provided in Table 1. Two additional nonviral guanine residues were added to the 5′ end of the LMT1 sequence to promote effective transcription by T7 RNA polymerase. The sequence of T68-1 LMT1, harboring 794 5′-terminal nts, was amplified via reverse transcription (RT)-PCR from the total RNA extracted from the *C. macrophylla* plants infected with CTV T68-1 using specific oligonucleotide primers and cloned into pUC19 under the T7 promoter using StuI and EcoRI. Sequences of the primers used for cloning are provided in Table 2. Two additional nonviral guanine residues were added to the 5′ end of the T68-1 LMT1 sequence to promote effective transcription by T7 RNA polymerase.

### 2.3. Generation of LMT1 RNA

To produce LMT1 RNAs for the SHAPE-MaP analysis, the respective RNAs were in vitro transcribed as described below. Briefly, each pUC19 LMT1 plasmid was linearized using EcoRI (New England Biolabs, Ipswich, MA, USA), which cuts the plasmid to generate a template for LMT1 with the correct 3′-terminal nucleotide without any additional nonviral bases. The linearized plasmid was then purified using the QIAquick^®^ PCR purification kit according to the manufacturer’s instructions (QIAGEN, Germantown, MD, USA). A total of 50 ng of linearized template was set aside for gel analysis. Following purification, the plasmid template was used for in vitro transcription using the mMESSAGE mMACHINE™ T7 Transcription kit following the manufacturer’s instructions (Thermo Fisher Scientific, Waltham, MA, USA). One modification was made to the procedure as LMT1 was not shown to be capped, so a different rNTP mix without a cap was used (New England BioLabs, Ipswich, MA, USA). Then, 1 μg of linearized plasmid was incubated for 2 h at 37 °C in a total volume of 20 μL. A total of 1 μL of the RNA and DNA mixture was set aside to be run on a gel. The newly transcribed RNA was then treated with 1 μL of TURBO™ DNase at 37 °C for 15 min to remove the DNA template (Thermo Fisher Scientific, Waltham, MA, USA). Following this, 1 μL of the DNase-treated RNA template was set aside to run on a gel. The RNA was then cleaned up using the ZYMO RNA Clean & Concentrator kit (ZYMO, Irvine, CA, USA), and 1 μL of purified RNA was kept for the analysis on a gel. A 1% agarose gel was used to evaluate each step of the RNA generation process. The RNA was then quantified following the manufacturer’s instructions by Qubit™ fluorometer, using a broad-range RNA assay (Thermo Fisher Scientific, Waltham, MA, USA).

### 2.4. RNA Folding, Modification, and Reverse Transcription

To fold, modify, and profile the RNA, a procedure described by Smola et al. (2015) [31] was performed with some modifications. In brief, 5 pmol of the transcribed RNA was added to 12 μL of sterile water. The RNA was then incubated at 95 °C for two minutes to denature it. Next, the RNA was immediately placed on ice for two minutes. Following this, 6 μL of 3.3× folding buffer (333 mM HEPES (pH 8.0), 333 mM NaCl, 33 mM MgCl_2_) was added to the RNA and mixed by pipetting, after which the RNA was allowed to fold at 25 °C for 20 min. A temperature of 25 °C was used instead of 37 °C for the RNA folding, since this is more biologically relevant for plant virus RNA. Then, 9 μL of the folded RNA was run on a 5% native polyacrylamide gel (10× TBE buffer (1 M Tris, 0.9 M boric acid, 0.01 M EDTA), 30% acrylamide and bis acrylamide solution, TEMED, and 10% ammonium persulfate solution) and electrophoresed for 105 min at 120 V on ice. The gel was then stained with ethidium bromide for 5 min and visualized under UV light.

After folding, the RNA was modified by mixing 9 μL of the folded RNA with either 1 μL of 200 mM 2-methylnicotinic acid imidazolide (NAI) for the positive reaction or with 1 μL of dimethyl sulfoxide (DMSO) for the unmodified control reaction. The modification reaction was allowed to proceed for 15 min at 25 °C, followed by placing it on ice. The modified RNA was then purified using the Monarch RNA purification kit according to the manufacturer’s instructions (New England Biolabs, Ipswich, MA, USA). The RNA was then quantified by Qubit™ fluorometer, using a broad-range RNA assay (Thermo Fisher Scientific, Waltham, MA, USA).

The RT reaction was carried out as described. A 5× RT buffer (250 mM Tris-HCl (pH 8.3), 375 mM KCl, 15 mM MnCl_2_) was freshly prepared. The two different RT primers tested to confirm the ability to transcribe the entire modified RNA are shown in Table 1. A total of 1 pmol of RNA sample was combined with 1 μL of 10 mM dNTPs (New England BioLabs, Ipswich, MA, USA) and 1 μL of the 2 μM primers shown in Table 1. This mixture was incubated at 65 °C for 5 min in a thermocycler and then quickly cooled on ice. Then, 4 μL of 5× RT buffer, 2 µL of 0.1 M DTT, and 1 µL of SUPERase-IN™ (RNase inhibitor, Thermo Fisher Scientific, Waltham, MA, USA) were added before incubation at 25 °C for 2 min. Finally, 1 μL of Superscript II reverse transcriptase (200 U/μL; Thermo Fisher Scientific, Waltham, MA, USA) was added to each tube. The samples were incubated in a thermocycler using the following conditions: 25 °C for 10 min, 42 °C for 3 h, 70 °C for 15 min, followed by holding at 12 °C. The cDNA was then purified by Microspin™ G-50 columns (Cytiva, Marlborough, MA, USA).

### 2.5. SHAPE-MaP Library Construction and Sequencing

After the RT, two separate PCR reactions were performed to generate the SHAPE-MaP library. Primers for each reaction can be found in Table 1. The first PCR was performed as follows: A total of 5 μL of purified cDNA was combined with 10 μL of Q5 reaction buffer, 1 μL of dNTPs (10 mM each), Step 1 forward and reverse primers (10 μM each), 0.5 μL of Q5 hot-start DNA polymerase (2 U/μL), and nuclease-free water to a final volume of 50 μL. Then, the mixture was incubated in a thermocycler using the following conditions: 98 °C for 30 s, followed by 5 cycles of 98 °C for 10 s, 65 °C for 30 s, 72 °C for 20 s, followed by 2 min of extension at 72 °C, and then held at 12 °C. The reaction was then purified with the PureLink™ PCR Micro kit and eluted in 10 μL of nuclease-free water (Thermo Fisher Scientific, Waltham, MA, USA).

The second PCR was performed as follows: A total of 10 μL of purified PCR product was combined with 10 μL of Q5 reaction buffer, 1 μL of dNTPs (10 mM each), Step 2 forward and reverse primers (10 μM each), 0.5 μL of Q5 hot-start DNA polymerase (2 U/μL), and nuclease-free water to a volume of 50 μL. Then, the mixture was incubated in a thermocycler with the following conditions: 98 °C for 30 s, followed by 25 cycles of 98 °C for 10 s, 65 °C for 30 s, and 72 °C for 20 s, followed by 2 min of extension at 72 °C, and then held at 12 °C until the next step. The barcode-containing primers used for library generation are listed in Table 3.

The libraries were purified using Agencourt AMPure XP beads for clean-up (Beckman Coulter, Brea, CA, USA). The AMPure beads were allowed to reach room temperature and then vortexed thoroughly before use. A total of 45 μL of beads was added to each reaction and then pipetted to mix. The bead mixture was incubated for 5 min at room temperature and then placed on a magnetic stand until all the beads aggregated around the magnet. The supernatant was removed, and the bead pellet was washed with freshly prepared 80% ethanol three times. Excess ethanol was removed, and the beads were air-dried. The samples were removed from the magnetic stand, and 17 μL of nuclease-free water was added to resuspend the beads; these were allowed to incubate at room temperature for 2 min, placed back on the magnetic stand, and 15 μL of supernatant was transferred to a new tube.

The library concentration was evaluated via Qubit™ fluorometer, using a broad-range DNA assay (Thermo Fisher Scientific, Waltham, MA, USA), and the library fragment size was analyzed by the TapeStation Fragment Analyzer System (Agilent Technologies, Santa Clara, CA, USA). The first set of libraries with the T36 LMT1 RNA of 750 nts was sequenced on the Illumina MiSeq 2 × 250 platform at the University of Florida Interdisciplinary Center for Biotechnology Research (ICBR). The next set of libraries with the T36 and T68-1 LMT RNAs of 794 nts was sequenced on the Illumina MiSeq 2 × 300 platform at ICBR.

### 2.6. Generation of SHAPE Profiles and Structure Predictions

The NAI-treated RNA and the DMSO-treated control RNA were used for all comparisons. The resulting fastq files were trimmed to remove masked primer sequences, and two amplicons per sample were merged to cover the entire LMT1 sequence. All libraries were processed with the ShapeMapper2 pipeline to generate SHAPE reactivity profiles, and Superfold 1.0 was used to generate Shannon entropy profiles [33,37]. Structures were rendered with the RNAstructure program (version 6.4) [38], ViennaRNA RNAfold webserver (http://rna.tbi.univie.ac.at/cgi-bin/RNAWebSuite/RNAfold.cgi, accessed on 12 February 2025) [39,40], and the RNAcanvas web app (https://rna2drawer.app/, accessed on 12 February 2025) [41].

## 3. Results

### 3.1. Determination of the 3′ Ends of LMT1 RNAs Produced upon Infection with the T36 and T68-1 Variants of CTV

For lncRNAs, which do not encode polypeptides, RNA structure may be a key determinant of function. To identify regions of potential functional importance within LMT1 and compare those across CTV strains, one representative variant from each of the eight CTV genotypes (strains)—RB (FJ525433.1), T36 (AY170468.1), HA16-5 (GQ454870.1), S1 (KU589212.1), T68-1 (JQ965169.1), T30 (EU937520.1), VT (DQ151548.1), and T3 (KC525952.1)—was selected for in silico structural conservation predictions of their LMT1 RNAs.

To define the accurate nucleotide length of LMT1 RNAs to be used for comparison, we experimentally determined the 3′ termini of LMT1s from the T36 and T68-1 variants representing the T36 and T68 strains, respectively, using 3′ RACE on the RNA extracted from plants infected with the corresponding viruses. The 3′ end of T36 LMT1 had been previously mapped; however, the 3′ end of T68-1 LMT1 had not been resolved yet. In the study conducted by Ayllon et al. [20], the 3′ ends of the majority of clones harboring the T36 LMT1 sequence mapped to position 765 from the 5′ terminus of the T36 genomic RNA. In contrast, a study by Kang et al. [19] mapped T36 LMT1 to a maximum and most prevalent length of 747 nts, using pull-down of LMT1 RNA with the viral p33 protein (Figure 1B). Here, we performed another assessment of the 3′ terminus of T36 LMT1 RNA. The 3′ ends in the majority of clones (10 out of 19) mapped to nt 765, in agreement with previous findings by Ayllon et al. [20], but 4 out of 19 clones showed the 3′ terminus corresponding to nt position 793, indicating that T36 LMT1 RNA could have a longer length (Figure 1B).

Next, we performed 3′ RACE to determine the 3′ end of T68-1 LMT1 using *N. benthamiana* plants infected with a hybrid virus—engineered based on the T36 cDNA clone in which the region between nts 108 and 3012 was substituted with a cognate region from the T68-1 genome (CTV-L1L2T68; [36])—as well as citrus plants infected with wild-type T68-1. Infection of *N. benthamiana* plants with CTV could be achieved only by *Agrobacterium*-mediated infiltration of the infectious cDNA clones. Since that of T68-1 was not available, we used a cDNA clone of the above hybrid virus—which harbored most of the nucleotide sequence corresponding to LMT1 of T68-1 plus the downstream regions corresponding to the T68-1-specific controller element responsible for LMT1 production—for the inoculation of *N. benthamiana* plants. The RNA extracts from this laboratory host were used for 3′ RACE. The analysis was followed by 3′ RACE conducted using the RNA extracted from the T68-1-infected *C. macrophylla*. The results indicated that the maximum length of T68-1 LMT1 maps to 794 nts (Figure 1C). Most of the clones (12 out of 20) obtained using *N. benthamiana* infected with CTV-L1L2T68 mapped to nt 794 in the T68-1 genome. In comparison, 13 out of 27 clones analyzed using the T68-1-infected citrus plants mapped to nt 793, while 2 out of 27 clones mapped to nt 794 (Figure 1C). Based on the above results, we used the 5′-terminal 794 nts of each of the selected viral genomes for comparison since this reflected the maximum length found for either T36 or T68-1 LMT1 RNA.

### 3.2. LocARNA Prediction of the Conserved LMT1 Motifs Based on a Consensus from Eight CTV Strains

**Figure 2:**
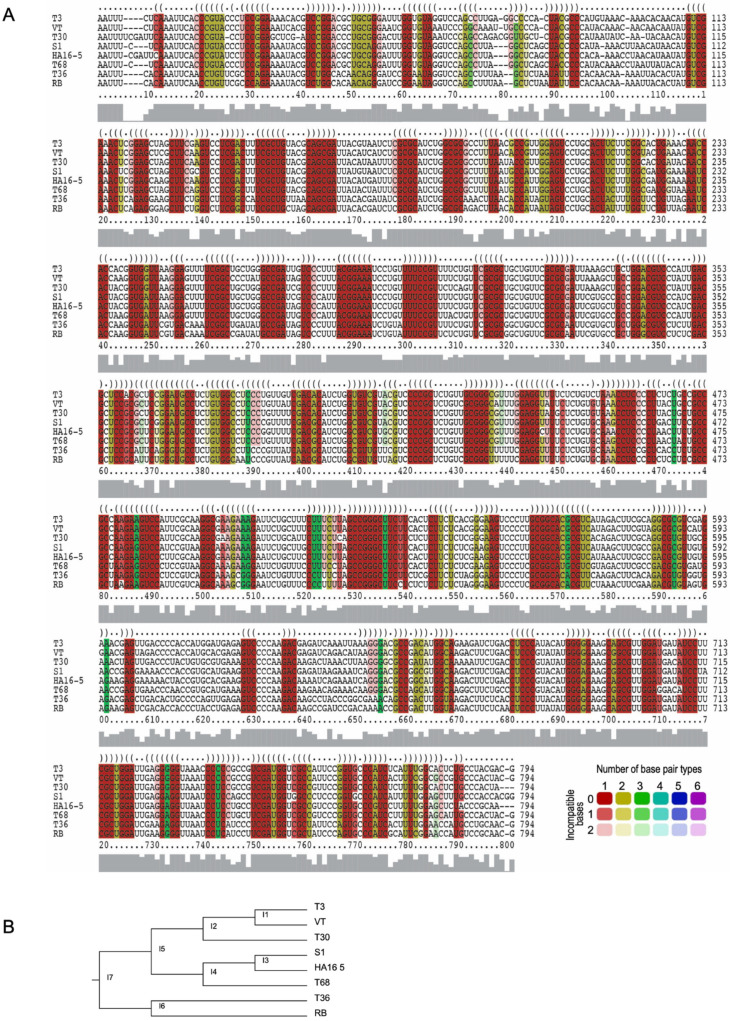
LMT1 structural alignment and structure-based phylogenetic analysis: (**A**) LocARNA structure-guided multiple sequence alignment of LMT1 RNAs produced by CTV variants. Representatives from eight CTV strains were used for the analysis. The names of the strains are indicated on the left side of the alignment. The GenBank accession numbers for the corresponding representative variants are provided in the text. Nucleotides are highlighted based on the number of compatible base-pair types, with lower-intensity colors indicating positions with some incompatible base pairs. (**B**) Phylogenetic tree showing the evolutionary relationships among LMT1 sequences. The tree was derived from the structure-guided alignment generated with locARNA.

The LMT1 sequences from the representative variants of the eight CTV strains were analyzed with the locARNA alignment webserver, which uses a simultaneous alignment and folding algorithm to predict conserved RNA structures [42]. As shown in Figure 2A, multiple regions of structural conservation were predicted across LMT1, depicted by the consensus dot-bracket notation across the top of the alignments. Different colors are used to highlight the respective regions, reflecting the number of base-pair types that were observed in each position, with a maximum of six (A:U, U:A, G:C, C:G, G:U, and U:G), while the color intensity refers to the conservation across the sequences. Nucleotides with green shading have sequence variations that preserve the base-pair interactions and lend additional support to the structures in the corresponding regions.

Previously, two structured regions of key functional importance have been identified at each terminus of LMT1 [17,43]. Despite ORF1a showing marked divergence between CTV strains, the 5′ untranslated region (UTR) was predicted to contain a conserved structure comprising two stem-loops separated by a small spacer [43]. The presence of these structures in the region comprising nts 10 to 98 of the consensus sequence is seen in the locARNA prediction obtained here (Figure 2A). Similarly, two stem-loop motifs involved in LMT1 production were found at the 3′ terminus of LMT1 earlier, using M-fold predictions and mutational analysis [17]. This element is located between nts 725 and 778 in the locARNA alignment (Figure 2A). Both sets of stem-loops show strong structural conservation across the eight sequences examined, even though the primary sequences within the 5′ UTRs are quite divergent. Approximately 17 additional stem-loop structures were predicted by the algorithm. While in silico predictions are a helpful first approximation, confirmation of an RNA structure requires validation in the system of interest.

To assess the accuracy of the locARNA predictions, we aimed to determine the LMT1 structures in two of the more distantly related CTV variants. The analysis of the phylogenetic relationships using the structure-driven locARNA alignment (Figure 2B) as well as the primary sequences of the complete viral genomes, the LMT1-encompassing regions, and the p23 gene-containing regions of the representative variants of the eight strains of CTV demonstrated that the sequences of T36 and T68-1 variants are among the most distantly related (Figure S1); therefore, further assessment of the LMT1 structure was done using the respective sequences of the latter variants.

### 3.3. SHAPE-MaP for the Analysis of the CTV LMT1 Structure

**Figure 3:**
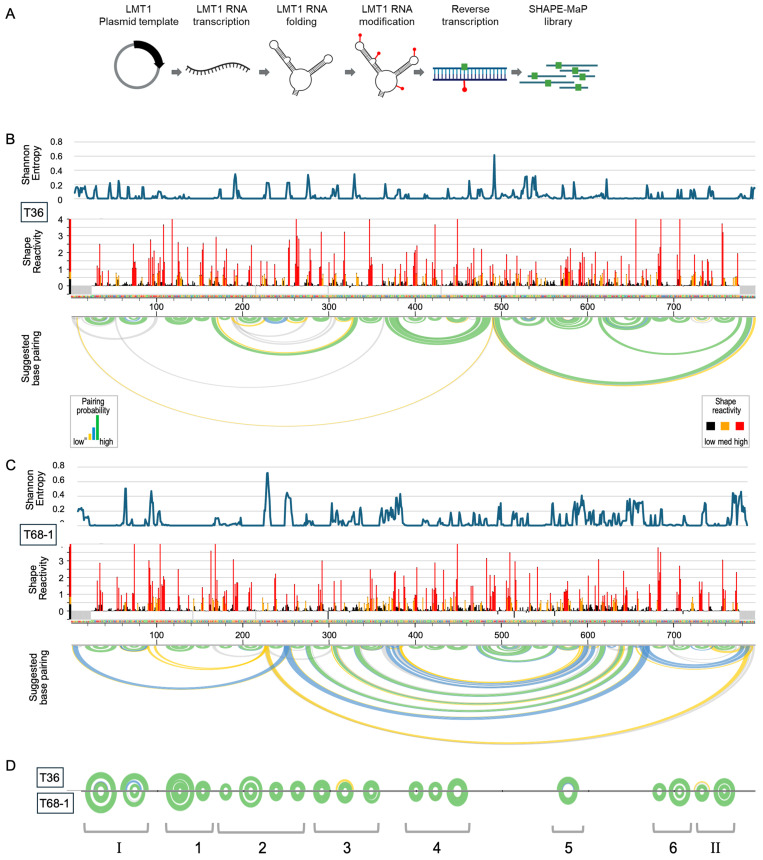
SHAPE-MaP analysis of LMT1 RNAs produced by the variants of two strains of CTV, T36 and T68: (**A**) Schematic overview of the SHAPE-MaP workflow used to probe the in vitro–produced LMT1 lncRNA. Created in BioRender. Spellman-Kruse, A. and Lawrence, A.J. (2026) https://biorender.com/6at623a. (**B**) Structural analysis of LMT1 of the T36 variant showing the Shannon entropy profile, SHAPE reactivity profile, and the SHAPE-guided base-pairing arc diagram. SHAPE reactivity values are color-coded to indicate nucleotide flexibility (red, high reactivity; orange, intermediate; black, low reactivity). In the arc diagram, base pairs are colored according to pairing confidence derived from SHAPE data (gray or yellow, low; blue, medium; green, high). (**C**) Structural analysis of LMT1 of the T68-1 variant showing the Shannon entropy profile, SHAPE reactivity profile, and the SHAPE-guided base-pairing arc diagram. (**D**) Comparison of arc diagrams highlighting conserved stem-loop structures shared between T36 (top) and T68-1 (bottom) LMT1s. I and II indicate previously studied elements, and 1–6 indicate newly identified stem-loop regions.

To experimentally validate the conserved LMT1 RNA structures, we adapted the SHAPE-MaP approach. SHAPE probing interrogates the ribose sugar backbone with an acylating reagent that reacts with unpaired nucleotides independently of the nucleobase identity. MaP relies on the ability of the reverse transcriptase to induce a mutation at the position of a SHAPE adduct, which is subsequently identified by sequencing (Figure 3A). This allows for the identification of more than one adduct per molecule and the structural changes within a single molecule. Given that LMT1 is coterminal with the CTV genome, we used an in vitro–transcribed LMT1 to avoid confounding effects from the genomic RNA.

Representative SHAPE-MaP reactivity profiles for T36 and T68-1 LMT1 RNAs were produced using the ShapeMapper2 pipeline and are depicted in the middle panels of Figure 3B and Figure 3C, respectively. Nucleotide positions shown in red and orange are highly or moderately reactive and indicate unpaired bases. Positions of low SHAPE reactivity, indicated in black, correspond to structured regions. The profiles were highly reproducible (Figures S2 and S3), and when we compared the two lengths of T36 LMT1—representing the longest isoform obtained in the 3′ RACE experiment of this study and the shorter isoform observed in the previous studies—we did not see significant differences in the global profile. Using SuperFold, which uses the ShapeMapper output to model RNA secondary structures, we obtained base-pairing prediction models for each isoform, shown in the lower panel in Figure 3B,C, with high-probability pairing interactions depicted with green arcs. Shannon entropies were also obtained from SuperFold and plotted in the upper panels. Regions with low Shannon entropy are more likely to form a single stable structure or to remain unpaired. Sequences with low SHAPE reactivity and low Shannon entropy are more likely to have well-defined RNA structures and are overrepresented with functional elements [44].

### 3.4. SHAPE-MaP Substantiates Stem-Loops Predicted Within the LMT1 Sequence

**Figure 4:**
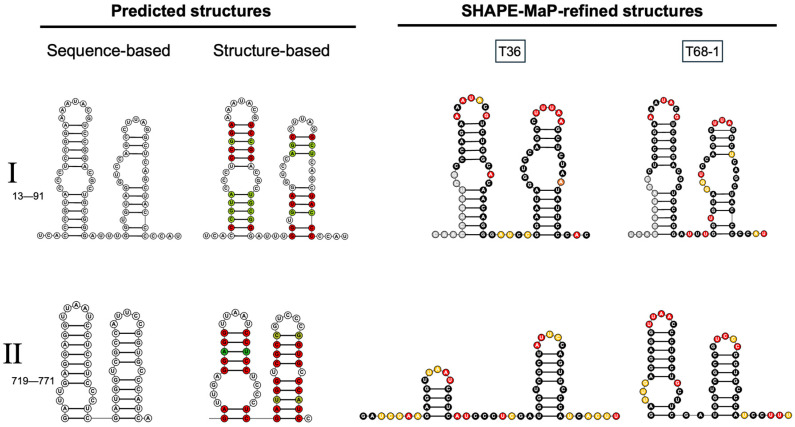
Predicted and SHAPE-refined structures of two previously identified functional elements of the 5′-proximal region of the CTV genome. Secondary structures of two previously identified elements, (**I**) (nts 13–91) and (**II**) (nts 719–771), that reside within the LMT1 region. Predicted structures derived from sequence-based and structure-guided alignments (generated using locARNA) are shown in the left-hand panel alongside the SHAPE-refined structures presented in the right-hand panel. The latter were obtained using SHAPE-MaP of LMT1s for T36 and T68-1. Colored bases in the structure-based stem-loops represent base-pair compatibility. SHAPE-reactive bases are highlighted in red for high reactivity (>0.85), orange for moderate reactivity (>0.4, <0.85), and black for low reactivity (<0.4) in the SHAPE-informed models.

We then reflected the base-pairing profiles of the T36 and T68-1 LMT1s to identify common predicted structures (Figure 3D), which also have low SHAPE reactivity and low Shannon entropy. Of the 19 predicted conserved stem-loop structures, only four have been previously identified, with two at each end of LMT1 (labeled as element I and II). The other elements were arbitrarily grouped into smaller sections for illustration purposes. The prediction consensus structures for the two known LMT1 elements are shown in Figure 4. The left-hand panels show the in silico structural predictions based on either primary sequence or structure-first alignments, which can be identical, as seen in the upper panels, or different (see lower panels). The right-hand columns show the SHAPE-refined structures for T36 and T68-1 LMT1 regions of these elements, with the SHAPE reactivity indicated.

In an earlier study, Gowda et al. (2003) [43] reported the MFOLD program-based prediction of the two stem-loops, referred to as SL1 and SL2, in the 107-nucleotide-long 5′ UTR of the CTV T36 genome and demonstrated the involvement of these structures in virus replication using mutational analysis. The predicted stem-loops were shown to be conserved in the 5′ UTR regions of variants of several other strains of CTV in the same study. Since the LMT1 sequence encompasses the UTR region, it was not surprising that both stem-loops were observed in our SHAPE-MaP analysis (element I; Figure 4, top row). For the structural element found at the 5′ end of LMT1 RNAs of both T36 and T68-1, light gray circles indicate regions where structural information is masked by the primers used during the amplicon amplification step, while orange and red circles indicate medium- and high-SHAPE reactivity, respectively. For both variants, the terminal loops and linker regions show good reactivity, supporting the predicted structures. It should be noted that the internal loops in element I lack the expected SHAPE reactivity profile, given the strong sequence- and structure-based predictions across CTV strains, indicating what should be single-stranded regions in the two internal loops. The internal loop of the first hairpin has two nucleotides with obscured reactivity (shown in gray) due to the position of the primers used for sequencing. The first internal loop of the T36 structure has one strongly reactive base on the 3′ side, while the T68-1 probing data do not indicate any reactive bases. The internal loop of the second hairpin is also less reactive than expected, particularly in T36, where only one nucleotide in the loop is moderately reactive. Gowda et al. (2003) [43] produced various mutants with alterations in the stem-loops of element I, manipulating the helix stems, the results of which provide high confidence that both hairpins exist and are critical for CTV replication [43]. Gowda et al. [43] did not alter the internal loops; however, the mutational analysis of the stems, along with the sequence- and structure-guided analyses coupled with the SHAPE reactivity data in our study, support the hairpins presented in Figure 4. Furthermore, the low SHAPE reactivity does not necessarily contradict the presence of the internal loops. SHAPE reports on the backbone flexibility, and unpaired nucleotides could be non-reactive if they are involved in tertiary structures, non-canonical base pairing, pseudoknots, or other rigid conformations. These interactions could shield the single-stranded regions from the SHAPE reagent, resulting in the low reactivity.

In another study, a set of stem-loops was also predicted in the region located between nts 719 and 775 of the CTV T36 genome [17]. SL1 and SL2 were shown to be important for the production of LMT1, as the authors assessed the function of these stem-loops through a number of mutations, including compensatory changes, deletions, and site-specific substitutions [17]. While SL2 tolerated structure- and sequence-based manipulation without disruption of LMT1 production, SL1 was shown to be the opposite [17]. All mutations in the SL1 region had deleterious effects on the production of LMT1 RNA [17]. In our study, we also detected two stem-loop structures referred to as element II in the corresponding region (Figure 4). In contrast to element I, element II, which encompasses the controller element at the 3′ end of LMT1, shows a difference between the primary sequence-based and structure-driven predictions of SL1. The experimental data obtained here most closely align with the structure-based prediction but present a shorter SLI and a linker before SLII, specifically, for LMT1 of T36 (Figure 4). These data support the presence of the controller element, but SHAPE-MaP adds a significant alteration to the predicted structures, emphasizing the importance of experimental verification to complement in silico predictions.

### 3.5. Newly Identified Structures from Consensus Structure-Based Prediction and SHAPE-MaP Analysis

**Figure 5:**
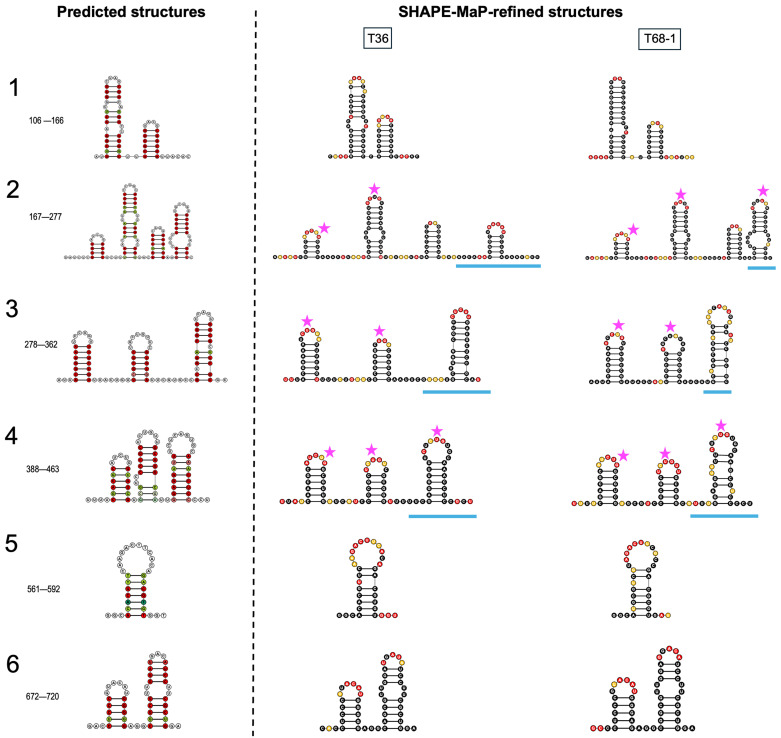
Structural alignment–based and SHAPE-refined structures of 15 newly identified stem-loops from the LMT1 region. Predicted structures derived from structure-guided alignments (generated using locARNA) are shown in the left-hand panel alongside the SHAPE-refined structures in the right-hand panel. The latter were obtained using SHAPE-MaP of LMT1s of T36 and T68-1. Colored bases in the structure-based stem-loops represent base-pair compatibility. SHAPE-reactive bases are highlighted in red for high reactivity (>0.85), orange for moderate reactivity (>0.4, <0.85), and black for low reactivity (<0.4) in the SHAPE-informed models. Regions (**1**–**6**) are arbitrarily defined for ease of discussion, and the corresponding nucleotide positions are indicated in the left-hand panel. Magenta stars indicate a common CUG motif in the loops. Blue underlines represent regions where SHAPE-derived structures differ from the structure-guided alignments.

Next, we compared the 15 stem-loop structures that we identified in the LMT1 sequences of the T36 and T68-1 variants using the structure-based locARNA predictions as the reference. We have arbitrarily separated these stem-loops into six predicted structure segments to facilitate the comparison, which are referred to here as segments 1 (nts 106–166), 2 (nts 167–277), 3 (nts 278–362), 4 (nts 388–463), 5 (nts 561–592), and 6 (nts 672–720) (Figure 5). As shown in Figure 5, SHAPE-MaP analysis supports the conservation of 12 of the 15 predicted stem-loops with significant variation in only three of the predicted structures indicated by the blue lines (segments 2, 3, and 4). Segment 1 consists of two hairpin loops, with small internal loops that are refined in the SHAPE-MaP structures for each virus variant. For segment 2, SHAPE-MaP structures inform the prediction with multiple reactive nts in the LMT1 RNA profiles of both T36 and T68-1 LMT1s, extending the unpaired, flexible region on either side of the shortened second stem-loop. Additionally, the fourth stem-loop is supported by the T68-1-derived LMT1 data but takes on a different conformation in that of T36. Segment 3 shows variation in the third stem-loop, with SHAPE reactivity data varying between the LMT1 RNAs of the two variants, indicating differing hairpin conformations. For segment 4, the SHAPE-MaP data indicate that the second stem-loop is shorter than in the conservation-based prediction, and that the third stem-loop structure varies between the two CTV variants. In both segments 5 and 6, SHAPE reactivity supports the structure conservation–based prediction.

Analysis of the terminal-loop sequences in the identified LMT1 stem-loops revealed a recurring CUG motif in multiple structures for both CTV variants. In T36 LMT1, 7 of 15 loops contain a CUG sequence, while 8 of 15 loops identified in LMT1 of T68-1 demonstrate the CUG pattern. Further comparison showed that seven of the CUG-containing loops are located at the same position in the LMT1 secondary structures of the two variants, showing positional conservation. The loops with the CUG motif vary in size from 4 to 9 nts and are clustered in the central portion of LMT1 within arbitrary segments 2, 3, and 4 (nts 167–463). In all cases, SHAPE-MaP reactivity profiles support the formation of these stem-loops. Given their broad conservation, the 15 newly identified stem-loops represent interesting elements for gaining insights into the functions of LMT1.

**Figure 6:**
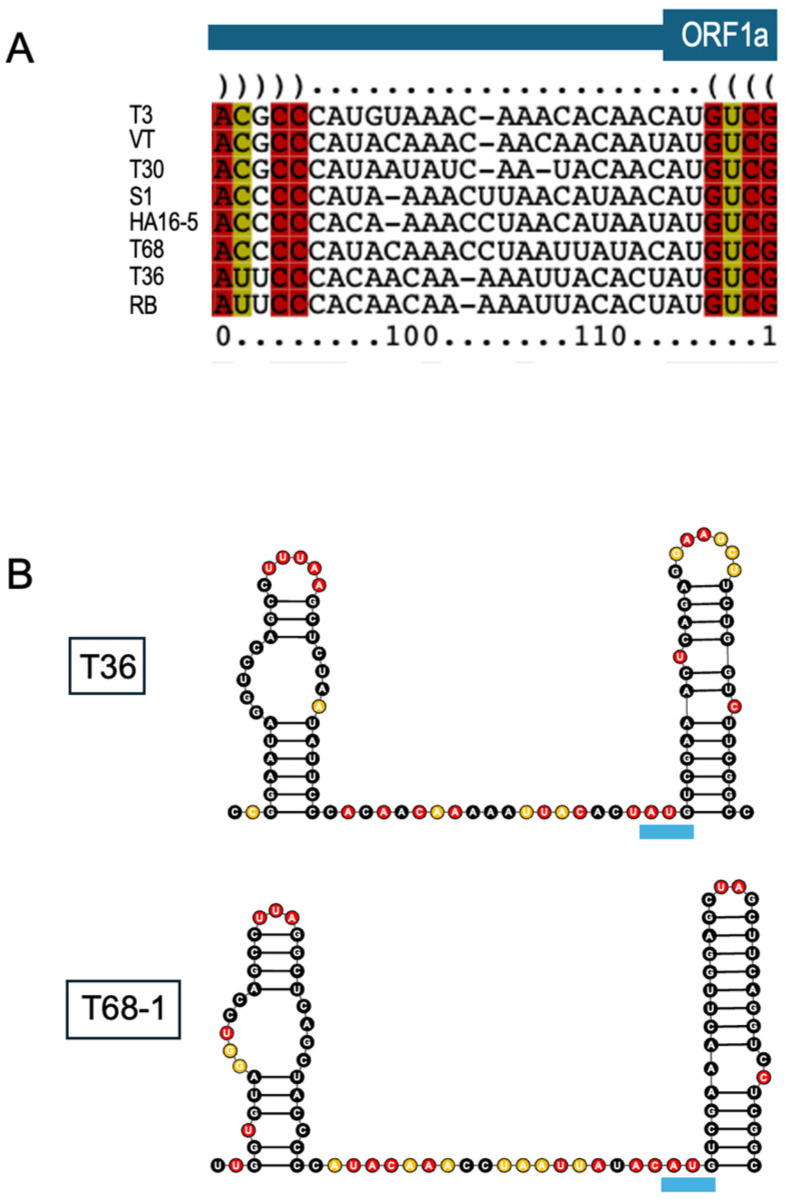
Structure analysis of a conserved region upstream of the ORF1a start codon: (**A**) LocARNA structure-guided multiple sequence alignment of the upstream region proximal to the ORF1a start codon. Representatives from eight CTV strains were used for the analysis. The names of the strains are indicated on the left side of the alignment. Bases are colored based on the number of compatible base-pair types. (**B**) SHAPE-MaP-informed secondary structures of the region upstream of the ORF1a start codon. SHAPE-reactive bases are highlighted in red for high reactivity (>0.85), orange for moderate reactivity (>0.4, <0.85), and black for low reactivity (<0.4). The blue underlines mark the start codon of ORF1a.

An additional interesting observation that arose from the SHAPE-MaP data relates to the conserved lack of structure immediately upstream of the start codon for ORF1a, a partial sequence of which is present in LMT1. As shown in Figure 6A, this region varies in length and primary sequence across variants of different strains, though all sequences harbor at least 50% adenine residues. The reactivity profiles across this region for T36 and T68-1 are depicted in Figure 6B and show high reactivity, speaking to the unpaired nature of the sequence. Viruses often use non-canonical modes of translation, including internal ribosome entry sites (IRESs). Typically, these are highly structured elements; however, it was demonstrated that turnip crinkle virus uses an IRES that is adenine-rich and unstructured to produce its coat protein [45]. The position of the adenine-rich unstructured region adjacent to CTV ORF1a is intriguing, and it may function in a similar manner. Given that LMT1 is non-coding, yet 5′-coterminal with the CTV genome, it is possible that this region allows LMT1 to sequester certain interacting factors away from the genome to influence the timing of replication and translation.

The combination of in silico prediction using conservation information paired with experimental RNA structure analysis of representative members of the two CTV strains has uncovered multiple new structures within LMT1 as well as a conserved flexible region adjacent to ORF1a. Future studies will be required to unravel the roles of these elements in LMT1-mediated pathways.

## 4. Discussion

LncRNAs are a diverse class of RNA molecules broadly defined as transcripts longer than 200 nts that lack canonical protein-coding capacity. Although initially regarded as transcriptional noise due to the low expression and detection rates, plus the generally low sequence conservation [44,46], lncRNAs are now recognized as important regulators of gene expression. Like messenger RNAs (mRNAs), lncRNAs may be capped, spliced, and polyadenylated, yet they function primarily through interactions with DNA, RNA, and proteins to modulate gene expression [47,48,49]. Consequently, lncRNAs have been implicated in numerous biological processes, including chromatin remodeling, transcriptional regulation, RNA splicing, and post-transcriptional control [50,51,52,53].

The functions of RNA molecules are closely linked to their structure. Viral RNAs, in particular, rely on specific secondary and tertiary structures such as stem-loops, pseudoknots, kissing loops, long-range RNA-RNA interactions, and dynamic conformational changes [54,55]. The architecture of viral RNA genomes mediates their interactions with proteins, RNAs, and other molecular partners to effectively control the dynamics of the viral infection cycle and host interactions [54,56,57,58]. Typically, crucial regulatory structures of RNAs are conserved, and thus identifying such conserved structural motifs—especially those maintained by compensatory mutations—provides a powerful means to pinpoint functionally relevant elements.

In this work, we applied SHAPE-MaP to the CTV lncRNA LMT1 to experimentally refine its secondary structure. This approach allowed us to validate predicted motifs and generate a SHAPE-informed secondary structure prediction that yields a framework for future functional studies. Importantly, this experimentally constrained model offers a basis for mutational analyses that preserve global RNA architecture while exploring the roles of individual motifs. This type of analysis has led to the identification of novel structures critical for viral fitness in many contexts, including structures found in the RNA molecules of human immunodeficiency virus (HIV), hepatitis C virus (HCV), Sindbis virus (SINV), and Dengue virus (DENV) [54]. For instance, whole-genome SHAPE comparisons of HIV-1 and two HIV progenitor strains (SIVcpzMB897 and SIVmac239) demonstrated conserved functional and species-specific elements [54,59]. Work on HCV identified different elements necessary for virus replication and infectious virus production, highlighting structural motifs with varied roles and involvement in different stages of virus infection [54,60,61]. In alphaviruses like SINV, structural elements are not well conserved across species, but SHAPE reactivity–supported structural elements in SINV demonstrated their critical role in virus fitness despite being non-conserved [54,62]. Finally, DENV2 genome SHAPE explorations revealed tertiary structure elements important for viral fitness and genome architecture [54,63,64]. These are a few of the many studies that have demonstrated that formerly uncharacterized RNA structures in viral genomes are essential across virus families.

In this study, the combination of in silico and SHAPE-MaP analyses provided the first high-resolution secondary structure maps of LMT1 lncRNAs from multiple CTV strains. Using structural conservation across eight strains of CTV, we identified 19 putative stem-loops, including two previously characterized elements at the 5′ and 3′ termini. We validated 15 novel structures through SHAPE-MaP for representatives of the T36 and T68 strains. The high degree of reproducibility of the SHAPE reactivity profiles and the corresponding low SHAPE, low Shannon Entropy highlight the robustness of the structural predictions. Principal to the goal of this study, several stem-loops are conserved across the divergent LMT1 RNAs produced by variants of both strains and include compensatory nucleotide changes to preserve structural elements. This suggests that the RNA structure in the LMT1 region, rather than a primary sequence alone, may be under selective pressure and is central to LMT1 function.

Several specific structural features identified in this study warrant further investigation. The repeated CUG motif within multiple terminal loops is of interest as it could indicate recurrent binding sites for host or viral RNA binding proteins, although it is equally plausible that its functional importance lies within the stem-loop size and structure itself over the sequence therein. In addition, the discovery of the highly SHAPE-reactive, adenine-rich, flexible region upstream of ORF1a raises the possibility of a regulatory element that could influence translation and the involved RNA–protein interactions, reminiscent of unstructured IRES elements in other viruses. Collectively, these findings not only expand our understanding of this lncRNA structure and function in the CTV infection but also provide a foundation for future mechanistic studies aimed at defining the roles of specific RNA motifs in CTV replication, gene expression, and virus–host interactions. An important point that should be addressed is the overlapping of the LMT1 sequence with both the CTV genomic RNA and the lncRNA LMT2. There is a possibility that some of the observed structures function at the level of the latter RNA molecules rather than in LMT1. Further in vivo analysis of the RNA structure of the corresponding regions would be important to discern their biological roles. This analysis might also reveal nucleotide modifications such as m^6^A (N^6^-methyladenosine) that can impact the structure.

Our findings register LMT1 among a growing class of functional viral lncRNAs and emphasize RNA structure as an avenue for exploring the mechanisms of action. By providing an experimentally supported structural framework for LMT1, this study provides the groundwork for future research aimed at defining the roles of RNA motifs and their functions in virus replication, host interactions, and CTV strain interactions. Finally, this work showcases structured RNA motifs, which could be implemented as targets for antiviral agents.

## Figures and Tables

**Table 1:** Primers used for the generation of plasmid constructs for SHAPE-MaP, reverse transcription, and library construction.

	Primer Name	Primer Sequence (5′-3′)
1	T36 LMT1 750 SHAPE Plasmid FWD	TAATACGACTCACTATAGGAATTTCACAAATTCAACCTGTTCGCCCAG
2	T36 LMT1 750 SHAPE Plasmid REV	CGGAATTCCATCGAGGGATGAGGATTAACCTCTTCG
3	T36 LMT1 794 SHAPE Plasmid REV	CGGAATTCGTTGCAGGCATGGTTCCAAAAGT
4	RT primer T36 LMT1 794	CGTTGCAGGCATGGTTCC
5	RT primer A T36 LMT1 750	CCATCGAGGGATGAGGATTAA
6	RT primer B T36 LMT1 400	GATGCGTTGATAACGGGAAGG
7	Step 1 FWD Set A T36 1–24 nt	GACTGGAGTTCAGACGTGTGCTCTTCCGATCTNNNNNAATTTCACAAATTCAACCTGTTCG
8	Step 1 REV Set A T36 380–400 nt	CCCTACACGACGCTCTTCCGATCT NNNNNGATGCGTTGATAACGGGAAGG
9	Step 1 FWD Set B T36 350–367 nt	GACTGGAGTTCAGACGTGTGCTCTTCCGATCTNNNNNTGACGCTCCGCATTCAG
10	Step 1 REV Set B T36 730–750 nt	CCCTACACGACGCTCTTCCGATCTNNNNNCCATCGAGGGATGAGGATTAA
11	Step 1 REV B T36 777–794 nt	CCC TAC ACG ACG CTC TTC CGA TCT NNN NN CGTTGCAGGCATGGTTCC
12	SHAPE Universal FWD	CAAGCAGAAGACGGCATACGAGAT[Barcode]GTGACTGGAGTTCAG AC
13	SHAPE Universal REV	AATGATACGGCGACCACCGAGATCTACACTCTTTCCCTACACGACGCTCTTCCG
14	T68 LMT1 794 SHAPE FWD Primer	TAATACGACTCACTATAGGAATTTCTCAAATTCACCTGTACCC
15	T68 LMT1 794 SHAPE REV Primer	CGGAATTCGTAGTGGGCAATGCTCC
16	T68 RT Primer 794	CGTAGTGGGCAATGCTCC
17	Step 1 FWD A T68 1–24 nt	GAC TGG AGT TCA GAC GTG TGC TCT TCC GAT CTN NNN N AATTTCTCAAATTCACCTGTACC
18	Step 1 REV A T68 380–400 nt	CCC TAC ACG ACG CTC TTC CGA TCT NNN NN GATGCGTCGAAAACAGGGAGA
19	Step 1 FWD B T68 350–367 nt	GAC TGG AGT TCA GAC GTG TGC TCT TCC GAT CTN NNN N TGACGCTCCGCGCTCTG
20	Step 1 REV B T68 777–794 nt	CCC TAC ACG ACG CTC TTC CGA TCT NNN NN CGTAGTGGGCAATGCTCC

**Table 2:** Primers used for the generation of LMT1 truncations, the T68 LMT1 plasmid, and RT-PCR detection.

	Primer Name	Primer Sequence (5′-3′)
1	T36 LMT1 FWD	AATTTCACAAATTCAACCTGTTCGC
2	T36 LMT1 366 REV Kpn1	ACGGTACCCTGAATGCGGAGCGTCAAGAGGAC
3	T36 LMT1 552 REV Kpn1	ACGGTACCCTTCCCTAGAGAAGCGAGAAGAGGC
4	T36 LMT1 675 REV Kpn1	ACGGTACCGGTGAGAAGTCTTACCAAGTCGGCTGTTT
5	T36 LMT1 750 REV Kpn1	ACGGTACCCCATCGAGGGATGAGG
6	T36 LMT1 765 REV Kpn1	ACGGTACCGCACTGGAATAGCGACCAT
7	T36 LMT1 794 REV Kpn1	ACGGTACCCGTTGCAGGCATGGTTC
8	T68 LMT1 FWD Stu1	AGAGGCCTAATTTCTCAAATTCACCTGTAC
9	T68 LMT1 750 REV Kpn1	ACGGTACCCCATCGAAGCAGGAGG
10	CP FWD	ATGGACGACGAAACAAAGAAATTG
11	PC REV	TCAACGTGTGTTGAATTTCCCAAG

**Table 3:** Barcoded primers used for SHAPE-MaP library construction.

	Primer Name	Primer Sequence (5′-3′)
1	Barcode Primer 1	CAA GCA GAA GAC GGC ATA CGA GAT TCGCCTTA GTG ACT GGA GTT CAG AC
2	Barcode Primer 2	CAA GCA GAA GAC GGC ATA CGA GAT CTAGTACG GTG ACT GGA GTT CAG AC
3	Barcode Primer 3	CAA GCA GAA GAC GGC ATA CGA GAT TTCTGCCT GTG ACT GGA GTT CAG AC
4	Barcode Primer 4	CAA GCA GAA GAC GGC ATA CGA GAT GCTCAGGA GTG ACT GGA GTT CAG AC
5	Barcode Primer 5	CAA GCA GAA GAC GGC ATA CGA GAT AGGAGTCC GTG ACT GGA GTT CAG AC
6	Barcode Primer 6	CAA GCA GAA GAC GGC ATA CGA GAT CATGCCTA GTG ACT GGA GTT CAG AC
7	Barcode Primer 7	CAA GCA GAA GAC GGC ATA CGA GAT GTAGAGAG GTG ACT GGA GTT CAG AC
8	Barcode Primer 8	CAA GCA GAA GAC GGC ATA CGA GAT CCTCTCTG GTG ACT GGA GTT CAG AC
9	Barcode Primer 9	CAA GCA GAA GAC GGC ATA CGA GAT AGCGTAGC GTG ACT GGA GTT CAG AC
10	Barcode Primer 10	CAA GCA GAA GAC GGC ATA CGA GAT CAGCCTCG GTG ACT GGA GTT CAG AC
11	Barcode Primer 11	CAA GCA GAA GAC GGC ATA CGA GAT TGCCTCTT GTG ACT GGA GTT CAG AC
12	Barcode Primer 12	CAA GCA GAA GAC GGC ATA CGA GAT TCCTCTAC GTG ACT GGA GTT CAG AC
13	Barcode Primer 13	CAA GCA GAA GAC GGC ATA CGA GAT TCATGAGC GTG ACT GGA GTT CAG AC
14	Barcode Primer 14	CAA GCA GAA GAC GGC ATA CGA GAT CCTGAGAT GTG ACT GGA GTT CAG AC
15	Barcode Primer 15	CAA GCA GAA GAC GGC ATA CGA GAT TAGCGAGT GTG ACT GGA GTT CAG AC
16	Barcode Primer 16	CAA GCA GAA GAC GGC ATA CGA GAT GTAGCTCC GTG ACT GGA GTT CAG AC
17	Barcode Primer 17	CAA GCA GAA GAC GGC ATA CGA GAT TACTACGC GTG ACT GGA GTT CAG AC
18	Barcode Primer 18	CAA GCA GAA GAC GGC ATA CGA GAT AGGCTCCG GTG ACT GGA GTT CAG AC
19	Barcode Primer 19	CAA GCA GAA GAC GGC ATA CGA GAT GCAGCGTA GTG ACT GGA GTT CAG AC
20	Barcode Primer 20	CAA GCA GAA GAC GGC ATA CGA GAT CTGCGCAT GTG ACT GGA GTT CAG AC
21	Barcode Primer 21	CAA GCA GAA GAC GGC ATA CGA GAT GAGCGCTA GTG ACT GGA GTT CAG AC
22	Barcode Primer 22	CAA GCA GAA GAC GGC ATA CGA GAT CGCTCAGT GTG ACT GGA GTT CAG AC
23	Barcode Primer 23	CAA GCA GAA GAC GGC ATA CGA GAT GTCTTAGG GTG ACT GGA GTT CAG AC
24	Barcode Primer 24	CAA GCA GAA GAC GGC ATA CGA GAT ACTGATCG GTG ACT GGA GTT CAG AC
25	Barcode Primer 25	CAA GCA GAA GAC GGC ATA CGA GAT TAGCTGCA GTG ACT GGA GTT CAG AC
26	Barcode Primer 26	CAA GCA GAA GAC GGC ATA CGA GAT GACGTCGA GTG ACT GGA GTT CAG AC

## Data Availability

Data are contained within the article and Supplementary Materials.

## Supplementary Materials

The following supporting information can be downloaded at: https://www.mdpi.com/article/10.3390/v18040470/s1, Figure S1: Phylogenetic analysis of (A) whole RNA genome sequences for CTV strains aligned in Clustal-omega, (B) LMT1 (794 nts) RNA sequences aligned using Clustal-omega, (C) LMT1 (794 nts) RNA sequences aligned via locARNA structural alignment, (D) p23 subgenomic RNA sequences aligned using Clustal-omega, and (E) p23 subgenomic RNA sequences aligned via locARNA structural alignment; Figure S2: Reproducibility of the SHAPE-MaP analysis of LMT1 of T36; Figure S3: Reproducibility of the SHAPE-MaP analysis of LMT1 of T68-1.

## References

[1] Aknadibossian V., Bar-Joseph M., Catara A.F., Cook G., Donovan N., Hajeri S., Licciardello G., Vidalakis G., Wulff N.A., Folimonova S.Y. (2026). Citrus Tristeza Virus: From Devastating Epidemics to Effective Management in Citrus-Producing Regions Around the World. Plant Dis..

[2] Sun Y., Yokomi R.K., Folimonova S.Y. (2024). Citrus Tristeza Virus: A Century-Long Challenge for the World’s Citrus Industries. Ann. Appl. Biol..

[3] Dawson W.O., Bar-Joseph M., Garnsey S.M., Moreno P. (2015). Citrus Tristeza Virus: Making an Ally from an Enemy. Annu. Rev. Phytopathol..

[4] Ghosh D.K., Kokane A., Kokane S., Mukherjee K., Tenzin J., Surwase D., Deshmukh D., Gubyad M., Biswas K.K. (2022). A Comprehensive Analysis of Citrus Tristeza Variants of Bhutan and Across the World. Front. Microbiol..

[5] Harper S.J. (2013). Citrus Tristeza Virus: Evolution of Complex and Varied Genotypic Groups. Front. Microbiol..

[6] Yokomi R., Selvaraj V., Maheshwari Y., Chiumenti M., Saponari M., Giampetruzzi A., Weng Z., Xiong Z., Hajeri S. (2018). Molecular and Biological Characterization of a Novel Mild Strain of Citrus Tristeza Virus in California. Arch. Virol..

[7] Folimonova S.Y., Sun Y.-D. (2022). Citrus Tristeza Virus: From Pathogen to Panacea. Annu. Rev. Virol..

[8] Moreno P., Ambros S., Albiach-Martí M.R., Guerri J., Peña L. (2007). Citrus Tristeza Virus: A Pathogen That Changed the Course of the Citrus Industry. Mol. Plant Pathol..

[9] Folimonova S.Y. (2020). Citrus Tristeza Virus: A Large RNA Virus with Complex Biology Turned into a Valuable Tool for Crop Protection. PLoS Pathog..

[10] Dolja V.V., Karasev A.V., Koonin E.V. (1994). Molecular Biology and Evolution of Closteroviruses: Sophisticated Build-up of Large RNA Genomes. Annu. Rev. Phytopathol..

[11] Agranovsky A.A. (1996). Principles of Molecular Organization, Expression, and Evolution of Closteroviruses: Over the Barriers. Adv. Virus Res..

[12] Bar-Joseph M., Garnsey S.M., Gonsalves D. (1979). The Closteroviruses: A Distinct Group of Elongated Plant Viruses. Adv. Virus Res..

[13] Dolja V.V., Kreuze J.F., Valkonen J.P.T. (2006). Comparative and Functional Genomics of Closteroviruses. Virus Res..

[14] Karasev A.V. (2000). Genetic Diversity and Evolution of Closteroviruses. Annu. Rev. Phytopathol..

[15] Karasev A.V., Boyko V.P., Gowda S., Nikolaeva O.V., Hilf M.E., Koonin E.V., Niblett C.L., Cline K., Gumpf D.J., Lee R.F. (1995). Complete Sequence of the Citrus Tristeza Virus RNA Genome. Virology.

[16] Pappu H.R., Karasev A.V., Anderson E.J., Pappu S.S., Hilf M.E., Febres V.J., Eckloff R.M.G., McCaffery M., Boyko V., Gowda S. (1994). Nucleotide Sequence and Organization of Eight 3′ Open Reading Frames of the Citrus Tristeza Closterovirus Genome. Virology.

[17] Gowda S., Ayllón M.A., Satyanarayana T., Bar-Joseph M., Dawson W.O. (2003). Transcription Strategy in a Closterovirus: A Novel 5′-Proximal Controller Element of Citrus Tristeza Virus Produces 5′- and 3′-Terminal Subgenomic RNAs and Differs from 3′ Open Reading Frame Controller Elements. J. Virol..

[18] Peng C.W., Peremyslov V.V., Mushegian A.R., Dawson W.O., Dolja V.V. (2001). Functional Specialization and Evolution of Leader Proteinases in the Family *Closteroviridae*. J. Virol..

[19] Kang S.-H., Aknadibossian V., Kharel L., Mudiyanselage S.D.D., Wang Y., Folimonova S.Y. (2021). The Intriguing Conundrum of a Nonconserved Multifunctional Protein of Citrus Tristeza Virus That Interacts with a Viral Long Non-Coding RNA. Viruses.

[20] Ayllón M.A., Gowda S., Satyanarayana T., Dawson W.O. (2004). Cis-Acting Elements at Opposite Ends of the Citrus Tristeza Virus Genome Differ in Initiation and Termination of Subgenomic RNAs. Virology.

[21] Wierzbicki A.T., Blevins T., Swiezewski S. (2021). Long Noncoding RNAs in Plants. Annu. Rev. Plant Biol..

[22] Mattick J.S., Amaral P.P., Carninci P., Carpenter S., Chang H.Y., Chen L.-L., Chen R., Dean C., Dinger M.E., Fitzgerald K.A. (2023). Long Non-Coding RNAs: Definitions, Functions, Challenges and Recommendations. Nat. Rev. Mol. Cell Biol..

[23] Tycowski K.T., Guo Y.E., Lee N., Moss W.N., Vallery T.K., Xie M., Steitz J.A. (2015). Viral Noncoding RNAs: More Surprises. Genes Dev..

[24] Miller W.A., Shen R., Staplin W., Kanodia P. (2016). Noncoding RNAs of Plant Viruses and Viroids: Sponges of Host Translation and RNA Interference Machinery. Mol. Plant-Microbe Interact..

[25] Wang Y., Folimonova S.Y. (2023). Long Noncoding RNAs in Plant–Pathogen Interactions. Phytopathology.

[26] Blevins T., Rajeswaran R., Aregger M., Borah B.K., Schepetilnikov M., Baerlocher L., Farinelli L., Meins F., Hohn T., Pooggin M.M. (2011). Massive Production of Small RNAs from a Non-Coding Region of Cauliflower Mosaic Virus in Plant Defense and Viral Counter-Defense. Nucleic Acids Res..

[27] Flobinus A., Hleibieh K., Klein E., Ratti C., Bouzoubaa S., Gilmer D. (2016). A Viral Noncoding RNA Complements a Weakened Viral RNA Silencing Suppressor and Promotes Efficient Systemic Host Infection. Viruses.

[28] Che X., Piestun D., Mawassi M., Yang G., Satyanarayana T., Gowda S., Dawson W.O., Bar-Joseph M. (2001). 5′-Coterminal Subgenomic RNAs in Citrus Tristeza Virus-Infected Cells. Virology.

[29] Gowda S., Tatineni S., Folimonova S.Y., Hilf M.E., Dawson W.O. (2009). Accumulation of a 5′ Proximal Subgenomic RNA of Citrus Tristeza Virus Is Correlated with Encapsidation by the Minor Coat Protein. Virology.

[30] Kang S.-H., Sun Y.-D., Atallah O.O., Huguet-Tapia J.C., Noble J.D., Folimonova S.Y. (2019). A Long Non-Coding RNA of Citrus Tristeza Virus: Role in the Virus Interplay with the Host Immunity. Viruses.

[31] Smola M.J., Rice G.M., Busan S., Siegfried N.A., Weeks K.M. (2015). Selective 2′-Hydroxyl Acylation Analyzed by Primer Extension and Mutational Profiling (SHAPE-MaP) for Direct, Versatile and Accurate RNA Structure Analysis. Nat. Protoc..

[32] McGinnis J.L., Dunkle J.A., Cate J.H.D., Weeks K.M. (2012). The Mechanisms of RNA SHAPE Chemistry. J. Am. Chem. Soc..

[33] Siegfried N.A., Busan S., Rice G.M., Nelson J.A.E., Weeks K.M. (2014). RNA Motif Discovery by SHAPE and Mutational Profiling (SHAPE-MaP). Nat. Methods.

[34] El-Mohtar C.A., Dawson W.O. (2014). Exploring the limits of vector construction based on Citrus tristeza virus. Virology.

[35] Kang S.-H., Bak A., Kim O.-K., Folimonova S.Y. (2015). Membrane association of a nonconserved viral protein confers virus ability to extend its host range. Virology.

[36] Atallah O.O., Kang S.H., El-Mohtar C.A., Shilt T., Bergua M., Folimonova S.Y. (2016). A 5’-proximal region of the Citrus tristeza virus genome encoding two leader proteases is involved in virus superinfection exclusion. Virology.

[37] Busan S., Weeks K.M. (2018). Accurate Detection of Chemical Modifications in RNA by Mutational Profiling (MaP) with ShapeMapper 2. RNA.

[38] Reuter J.S., Mathews D.H. (2010). RNAstructure: Software for RNA Secondary Structure Prediction and Analysis. BMC Bioinform..

[39] Gruber A.R., Lorenz R., Bernhart S.H., Neuböck R., Hofacker I.L. (2008). The Vienna RNA Websuite. Nucleic Acids Res..

[40] Gruber A.R., Bernhart S.H., Lorenz R. (2015). The ViennaRNA Web Services. Methods Mol. Biol..

[41] Johnson P.Z., Simon A.E. (2023). RNAcanvas: Interactive Drawing and Exploration of Nucleic Acid Structures. Nucleic Acids Res..

[42] Will S., Joshi T., Hofacker I.L., Stadler P.F., Backofen R. (2012). LocARNA-P: Accurate Boundary Prediction and Improved Detection of Structural RNAs. RNA.

[43] Gowda S., Satyanarayana T., Ayllón M.A., Moreno P., Flores R., Dawson W.O. (2003). The Conserved Structures of the 5′ Nontranslated Region of *Citrus tristeza* Virus Are Involved in Replication and Virion Assembly. Virology.

[44] Boerneke M.A., Gokhale N.S., Horner S.M., Weeks K.M. (2023). Structure-First Identification of RNA Elements That Regulate Dengue Virus Genome Architecture and Replication. Proc. Natl. Acad. Sci. USA.

[45] May J., Johnson P., Saleem H., Simon A.E. (2017). A Sequence-Independent, Unstructured Internal Ribosome Entry Site Is Responsible for Internal Expression of the Coat Protein of Turnip Crinkle Virus. J. Virol..

[46] Pang K.C., Frith M.C., Mattick J.S. (2006). Rapid Evolution of Noncoding RNAs: Lack of Conservation Does Not Mean Lack of Function. Trends Genet..

[47] Struhl K. (2007). Transcriptional Noise and the Fidelity of Initiation by RNA Polymerase II. Nat. Struct. Mol. Biol..

[48] Ulitsky I., Bartel D.P. (2013). lincRNAs: Genomics, Evolution, and Mechanisms. Cell.

[49] Kopp F., Mendell J.T. (2018). Functional Classification and Experimental Dissection of Long Noncoding RNAs. Cell.

[50] Ziegler C., Kretz M. (2017). The More the Merrier—Complexity in Long Non-Coding RNA Loci. Front. Endocrinol..

[51] Zhang X., Wang W., Zhu W., Dong J., Cheng Y., Yin Z., Shen F. (2019). Mechanisms and Functions of Long Non-Coding RNAs at Multiple Regulatory Levels. Int. J. Mol. Sci..

[52] Dykes I.M., Emanueli C. (2017). Transcriptional and Post-Transcriptional Gene Regulation by Long Non-Coding RNA. Genom. Proteom. Bioinform..

[53] Vance K.W., Ponting C.P. (2014). Transcriptional Regulatory Functions of Nuclear Long Noncoding RNAs. Trends Genet..

[54] Boerneke M.A., Ehrhardt J.E., Weeks K.M. (2019). Physical and Functional Analysis of Viral RNA Genomes by SHAPE. Annu. Rev. Virol..

[55] Hendrix D.K., Brenner S.E., Holbrook S.R. (2005). RNA Structural Motifs: Building Blocks of a Modular Biomolecule. Q. Rev. Biophys..

[56] Rausch J.W., Sztuba-Solinska J., Le Grice S.F.J. (2018). Probing the Structures of Viral RNA Regulatory Elements with SHAPE and Related Methodologies. Front. Microbiol..

[57] Sola I., Mateos-Gomez P.A., Almazan F., Zuñiga S., Enjuanes L. (2011). RNA-RNA and RNA-Protein Interactions in Coronavirus Replication and Transcription. RNA Biol..

[58] Yuan X., Shi K., Young M.Y.L., Simon A.E. (2010). The Terminal Loop of a 3′ Proximal Hairpin Plays a Critical Role in Replication and the Structure of the 3′ Region of Turnip Crinkle Virus. Virology.

[59] Lavender C.A., Gorelick R.J., Weeks K.M. (2015). Structure-Based Alignment and Consensus Secondary Structures for Three HIV-Related RNA Genomes. PLoS Comput. Biol..

[60] Mauger D.M., Golden M., Yamane D., Williford S., Lemon S.M., Martin D.P., Weeks K.M. (2015). Functionally Conserved Architecture of Hepatitis C Virus RNA Genomes. Proc. Natl. Acad. Sci. USA.

[61] Pirakitikulr N., Kohlway A., Lindenbach B.D., Pyle A.M. (2016). The Coding Region of the HCV Genome Contains a Network of Regulatory RNA Structures. Mol. Cell.

[62] Kutchko K.M., Madden E.A., Morrison C., Plante K.S., Sanders W., Vincent H.A., Cruz Cisneros M.C., Long K.M., Moorman N.J., Heise M.T. (2018). Structural Divergence Creates New Functional Features in Alphavirus Genomes. Nucleic Acids Res..

[63] Dethoff E.A., Boerneke M.A., Gokhale N.S., Muhire B.M., Martin D.P., Sacco M.T., McFadden M.J., Weinstein J.B., Messer W.B., Horner S.M. (2018). Pervasive Tertiary Structure in the Dengue Virus RNA Genome. Proc. Natl. Acad. Sci. USA.

[64] Huber R.G., Lim X.N., Ng W.C., Sim A.Y.L., Poh H.X., Shen Y., Lim S.Y., Sundstrom K.B., Sun X., Aw J.G. (2019). Structure Mapping of Dengue and Zika Viruses Reveals Functional Long-Range Interactions. Nat. Commun..

