# Supplementary material for: SHAPE-MaP-Based Assessment of the Structure of Citrus Tristeza Virus Long Non-Coding RNA
Source: Viruses. 2026 Apr 16;18(4):470. doi: 10.3390/v18040470 (PMC13120261; doi:10.3390/v18040470)
Supplement: Supplementary file 1 [file viruses-18-00470-s001.zip › viruses-4228256-supplementary.pdf]

Supplemental figures

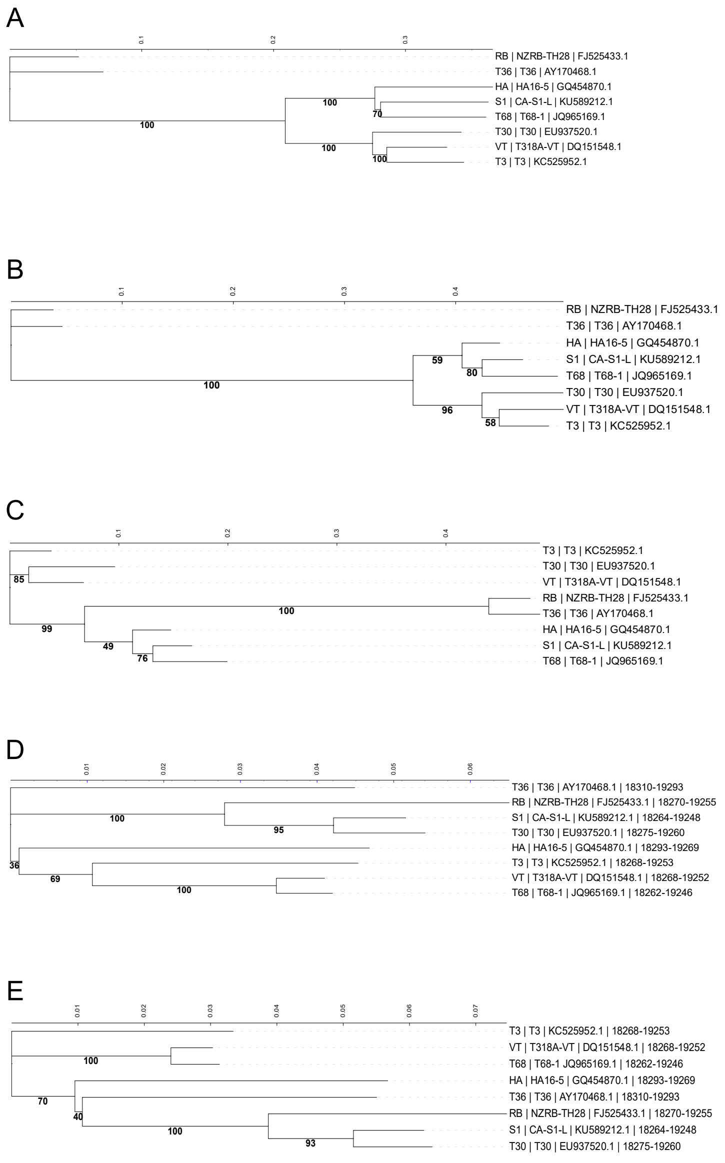

Figure S1. Phylogenetic analysis of A) whole RNA genome sequences for CTV strains aligned in Clustal-omega, B) LMT1 (794 nts) RNA sequences aligned using Clustal-omega, C) LMT1 (794 nts) RNA sequences aligned via locARNA structural alignment, D) p23 subgenomic RNA sequences aligned using Clustal-omega, and E) p23 subgenomic RNA sequences aligned via locARNA structural alignment. All phylogenies were constructed using the IQ-tree MFP algorithm with 1,000 bootstrap replicates.

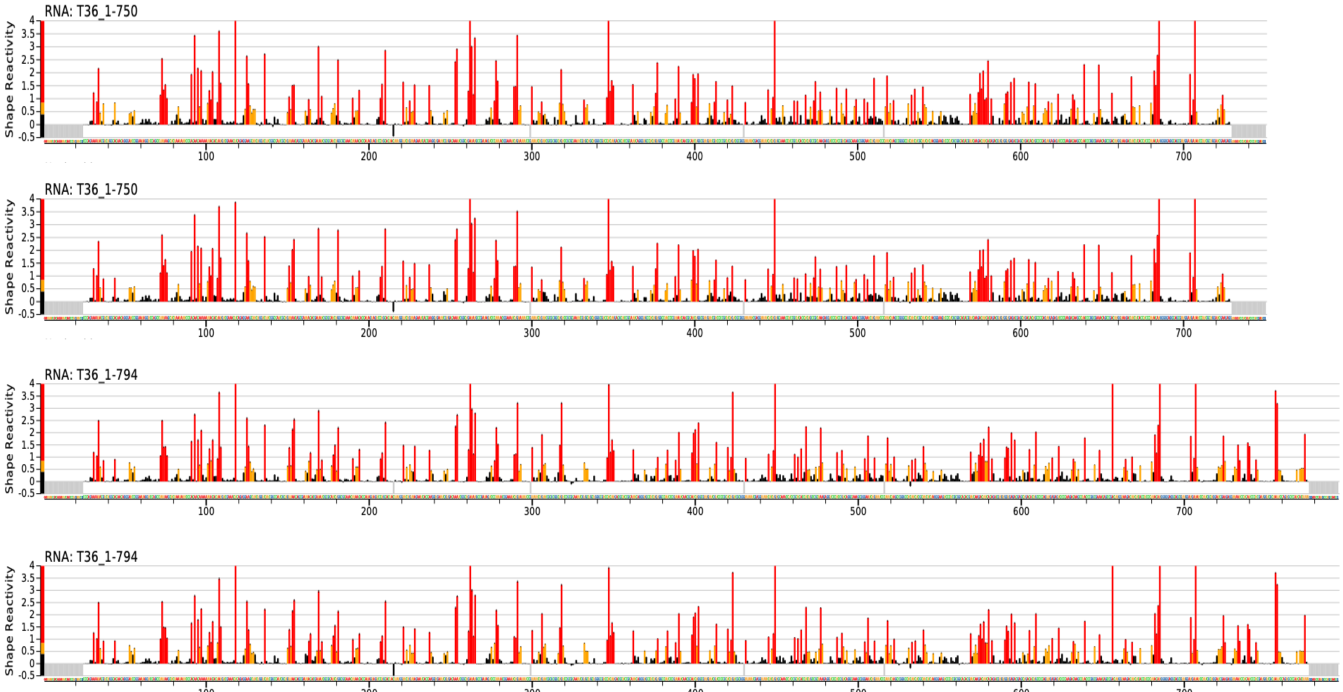

Figure S2. Reproducibility of the SHAPE-MaP analysis of LMT1 of T36. SHAPE-MaP data are highly reproducible among four sample of two lengths (750 and 794 nts) of T36 LMT1.

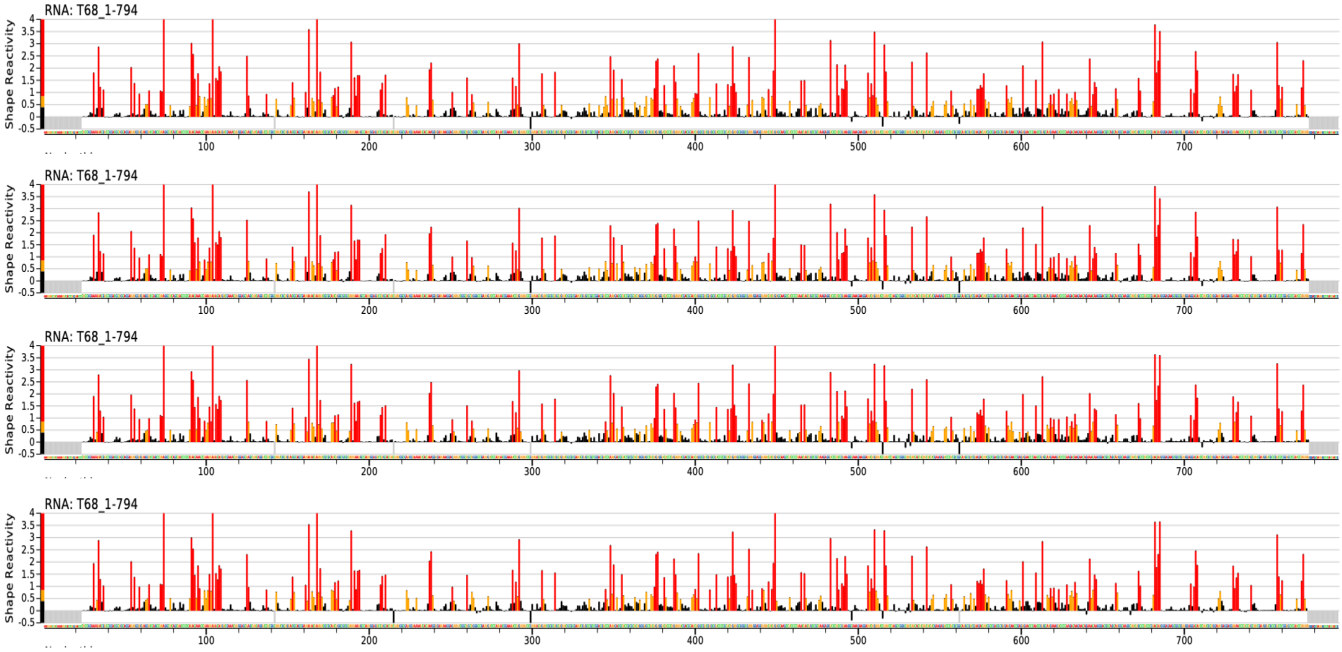

Figure S3. SHAPE-MaP reproducibility of LMT1 of T68-1. SHAPE-MaP data are highly reproducible among replicates (1-4 shown in order) of T68-1 LMT1.
